# Beam‐hardening correction in clinical x‐ray dark‐field chest radiography using deep‐learning‐based bone segmentation

**DOI:** 10.1002/mp.70422

**Published:** 2026-04-03

**Authors:** Lennard Kaster, Maximilian E. Lochschmidt, Anne M. Bauer, Tina Dorosti, Sofia Demianova, Thomas Koehler, Daniela Pfeiffer, Franz Pfeiffer

**Affiliations:** ^1^ Chair of Biomedical Physics, Department of Physics TUM School of Natural Sciences Technical University of Munich Garching Germany; ^2^ Munich Institute of Biomedical Engineering Technical University of Munich Garching Germany; ^3^ Institute for Diagnostic and Interventional Radiology, School of Medicine and Health, TUM Klinikum Technical University of Munich Munich Germany; ^4^ Institute of Advanced Study Technical University of Munich Garching Germany; ^5^ Philips Innovative Technologies Hamburg Germany

**Keywords:** chest radiography, dark‐field imaging, deep learning, segmentation, x‐ray imaging

## Abstract

**Background:**

Dark‐field radiography is a novel x‐ray imaging modality that provides complementary diagnostic information by visualising microstructural properties of lung tissue. Implemented via a Talbot–Lau interferometer integrated into a conventional x‐ray system, it permits simultaneous acquisition of perfectly registered attenuation and dark‐field radiographs. Clinical studies have shown that dark‐field radiography outperforms conventional radiography in diagnosing and staging pulmonary diseases, yet the polychromatic nature of medical x‐ray sources causes beam hardening and introduces structured artifacts, especially from ribs and clavicles.

**Purpose:**

To address the artificial dark‐field signal arising from beam‐hardening and thereby improve the reliability of clinical dark‐field chest radiography by suppressing bone‐induced artifacts.

**Methods:**

A segmentation‐based beam‐hardening correction (BHC) was developed that employs deep learning to segment ribs and clavicles and uses attenuation‐contribution masks derived from dual‐layer detector computed‐tomography data to refine the material distribution and estimate beam‐hardening effects. The rib segmentation network was trained on 196 chest radiographs with 49 validation images (VinDr‐RibCXR), and a clavicle network was trained on 56 images with 12 validation and 12 test cases. The trained models were applied to 174 dark‐field chest radiographs (51 chronic obstructive pulmonary disease, 86 COVID‐19, 37 healthy) and spectral CT scans from two patients; input data consisted of attenuation and dark‐field images and outputs were corrected dark‐field images and derived lung‐signal metrics.

**Results:**

The proposed method markedly reduced bone‐induced artifacts and improved the homogeneity of the lung dark‐field signal. In comparative analyses, the corrected images exhibited diminished structured cross‐talk between attenuation and dark‐field channels, enhancing both visual interpretation and quantitative consistency across cohorts.

**Conclusions:**

By combining deep‐learning‐based anatomical segmentation with material‐specific attenuation weighting, the proposed BHC suppresses the artificial dark‐field signal caused by polychromatic x‐ray spectra, leading to more reliable assessment of pulmonary microstructure in clinical dark‐field chest radiography.

## 1 INTRODUCTION

Dark‐field radiography is an emerging x‐ray imaging technique that enables visualization of micro‐structural properties of the sample under investigation that are otherwise inaccessible,^1^ such as the lung's alveolar structure.^2^, ^3^ Clinical studies have demonstrated improved diagnostic performance of dark‐field radiography for diagnosing and staging pulmonary diseases, such as COVID‐19, chronic obstructive pulmonary disease (COPD), and pneumothoraces when compared with conventional attenuation‐based radiography.^4^, ^5^, ^6^, ^7^, ^8^, ^9^ The dark‐field signal is generated from ultra‐small‐angle scattering (USAXS) at material interfaces, providing complementary information to the attenuation‐based conventional radiograph. Healthy pulmonary tissue generates a strong dark‐field signal due to its numerous alveoli, representing scattering air–tissue interfaces.^4^, ^10^ Pathological changes such as alveolar destruction, inflammation, or masses reduce these interfaces, resulting in a diminished dark‐field signal.^3^, ^4^, ^5^, ^8^, ^11^, ^12^, ^13^ In contrast, it was shown that non‐ or coarser‐microstructured tissues such as bone and soft tissue contribute minimally to the dark‐field signal,^14^ making the modality highly specific to pulmonary microstructure.

To detect the USAXS with clinical x‐ray sources, as typically employed in radiography and CT systems, a Talbot–Lau interferometer is positioned within the beam path.^15^, ^16^ The Talbot–Lau interferometer, comprising three gratings denoted as $G_{0}$, $G_{1}$, and $G_{2}$, allows for the simultaneous extraction of attenuation, differential phase‐contrast, and dark‐field signals.

The source grating $G_{0}$ enables the utilization of a conventional x‐ray source with a spot size significantly larger than the grating periods by converting the extended focal spot into an array of individually coherent line sources. This spatial coherence is required for the Talbot effect exploited by the phase grating $G_{1}$. The phase grating $G_{1}$ imprints a periodic phase modulation of the x‐ray wavefront, generating a periodic intensity self‐image pattern via the Talbot effect at fractional Talbot distance.^17^ However, the resolution of clinical flat‐panel detectors is insufficient to directly resolve this fine intensity pattern. Therefore, an analyzer grating $G_{2}$, with a period matching the intensity pattern, is positioned directly in front of the detector to sample the fine intensity pattern and convert variations in fringe visibility and phase into measurable intensity modulations.

Accordingly, the attenuation signal is encoded in a reduction of the mean detected intensity, as observed in conventional radiographs and CT scans. The dark‐field signal is encoded in a reduction of the visibility of the intensity pattern, affecting its relative amplitude or contrast and reflecting USAXS from unresolved microstructural interfaces. The phase shift of the pattern represents the differential phase‐contrast signal. By capturing different relative grating positions for each pixel, the attenuation, dark‐field, and (differential) phase‐contrast signals can be simultaneously extracted for each pixel.^16^ This capability enhances the potential of the Talbot–Lau interferometer in clinical x‐ray dark‐field chest radiography due to the perfectly spatially and temporally registered complementary information.

It is well‐known from conventional x‐ray radiographs and CT scans that strongly attenuating samples can cause artifacts impairing image quality and diagnostic accuracy.^18^ Although x‐ray attenuation does not directly affect the dark‐field signal itself, attenuation can still engender artifacts known as beam‐hardening‐induced dark‐field signals. Since the visibility—and thus the dark‐field signal—is dependent on the x‐ray beam spectrum, dark‐field radiography is similarly susceptible to beam hardening caused by attenuating samples.^19^, ^20^, ^21^ In this study, we present a fully automated beam‐hardening correction (BHC) framework for clinical dark‐field chest radiography, based on deep‐learning‐derived anatomical segmentation. The method leverages rib and clavicle segmentations from a U‐Net to generate spatially adaptive attenuation contribution maps. These maps are then used to correct beam‐hardening‐induced dark‐field artifacts. We apply and evaluate the approach on a multigroup clinical dataset comprising healthy subjects and subjects with COPD and COVID‐19, using both qualitative visual assessment and quantitative metrics to assess artifact suppression and signal homogeneity. Although evaluated retrospectively in this study, the correction algorithm has already been integrated into the clinical dark‐field scanner and is currently being assessed in ongoing human studies.

## 2 BACKGROUND

### 2.1 Mathematical description

As mentioned in Section 1, the Talbot–Lau interferometer not only enables the conventional‐attenuation‐based imaging, but also provides access to the dark‐field signal, which additionally offers complementary information about the microstructural composition of the irradiated sample. Mathematically, both signal channels can be expressed by Equation (1) for the transmission T(E) and by Equation (2) for the dark‐field signal D(E).

(1) $$\begin{array}{cc} & T\left(E\right)=\frac{S(E)}{S_{0}\left(E\right)}=\exp\left[-\int_{0}^{z_{0}}\mu \left(z{\hat{e}}_{z},E\right)\;dz\right] \end{array}$$

(2) $$\begin{array}{cc} & D\left(E\right)=-\ln\left(\frac{V(E)}{V_{0}\left(E\right)}\right)=c_{D}\left(E\right)\int_{0}^{z_{0}}\varepsilon \left(z{\hat{e}}_{z},E\right)\;dz \end{array}$$

Here, $\mu (z{\hat{e}}_{z},E)$ describes the energy‐dependent attenuation of the transmitted x‐ray beam, whereas $\varepsilon (z{\hat{e}}_{z},E)$ captures the corresponding visibility loss induced by ultra‐small‐angle x‐ray scattering from subresolution microstructure.^1^, ^2^, ^22^, ^23^, ^24^ The unit vector ${\hat{e}}_{z}$ denotes the direction from the x‐ray source to the corresponding detector pixel. The constant $c_{D}$ is determined by the grating parameters and the geometric configuration of the setup. The visibility during the sample scan is denoted by V(E), while $V_{0}\left(E\right)$ refers to the visibility in the reference scan without a sample in the beam path. The effective source intensity is given by $S_{0}\left(E\right)=\psi_{0}\left(E\right)\cdot R\left(E\right)$, where $\psi_{0}\left(E\right)$ represents the x‐ray intensity and R(E) is the energy‐dependent detector response function.

For a polychromatic x‐ray source, the attenuation $A_{p}$, visibility $V_{p}$, and dark‐field signal $D_{p}$ are mathematically expressed by Equations (3)–(5)^21^:

(3) $$\begin{array}{cc} & A_{p}=-\ln\left(\frac{\int_{0}^{\infty }S\left(E\right)\;dE}{\int_{0}^{\infty }S_{0}\left(E\right)\;dE}\right) \end{array}$$

(4) $$\begin{array}{cc} & V_{p}=\frac{\int_{0}^{\infty }V\left(E\right)\;S\left(E\right)\;dE}{\int_{0}^{\infty }S\left(E\right)\;dE} \end{array}$$

(5) $$\begin{array}{cc} & D_{p}=-\ln\left(\frac{V_{p}}{V_{\text{0,p}}}\right). \end{array}$$

### 2.2 Beam‐hardening‐induced dark‐field signal

The polychromatic spectrum of medical x‐ray sources and the associated energy‐dependent interactions with matter cause the x‐ray spectrum to be hardened along the beam by the object under examination (3). This results in a nonlinear relationship between measured attenuation and object thickness, a common issue in CT.^18^ Although the dark‐field signal is based solely on USAXS, which is independent of the attenuation, the spectral distortions induce an indirect effect. Specifically, the beam hardening of the spectrum $S_{0}\left(E\right)$ leads to an artificial dark‐field signal—commonly referred to as beam‐hardening‐induced dark‐field signal—due to the energy dependency of the visibility V(E) and $\varepsilon (z{\hat{e}}_{z},E)$, as described in Equation (4).^1^, ^19^, ^21^ Note that the influence of beam hardening on the dark‐field signal also depends on the x‐ray spectrum used and the setup parameters and geometry.^21^

Since beam hardening of the x‐ray spectrum does not convey information about the microstructural composition of the irradiated material, it is desired to remove this artificial signal—particularly pronounced at rib structures and manifesting as a rib‐induced step in the dark‐field signal—by applying an appropriate BHC.

If only a single type of attenuator is present, beam hardening can be accurately corrected through calibration measurements.^25^, ^26^ Additionally, it is possible to narrow the source spectrum by applying stronger prefiltration.^19^ However, this approach reduces flux and yields only a modest improvement in beam hardening effects. In chest x‐ray imaging, however, these approaches are not applicable, as two different types of attenuators—bone and soft tissue—are present. The key difficulty arises from the fact that the precise composition of bone and soft tissue within the thorax region is unknown. Therefore, a fast and effective method that has shown improvements in image quality in this context is the weighted single‐LUT approach.^27^ In this method, calibration curves were acquired for aluminum and water as surrogates for bone and soft tissue. These were then combined into a single look‐up table (LUT) using a weighting factor, which significantly reduced the rib‐induced step in the dark‐field image. However, in regions of very high attenuation, this approach leads to overcorrection of the dark‐field signal. Defining a global weighting factor depends therefore on the specific clinical question or requires a compromise between minimizing the rib artifact and avoiding overcorrection.

Building upon the single‐LUT approach, the present work takes a significant step forward by implementing a deep learning‐based BHC. This method addresses both the issue of overcorrection and the limitations associated with manually selecting a global weighting factor.

## 3 MATERIALS AND METHODS

### 3.1 Setup

**FIGURE 1:**
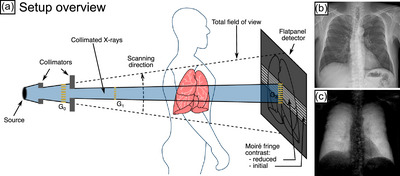
Schematic of the clinical dark‐field chest radiography system. The clinical dark‐field radiography system integrates a conventional x‐ray source, collimators, and a flat panel detector with a Talbot–Lau interferometer comprising a source grating $G_{0}$, phase grating $G_{1}$, and analyzer grating $G_{2}$. This interferometer induces an interference pattern with moiré fringes on the detector plane. By acquiring a series of exposures at varying phase steps of the fringe pattern, both attenuation‐based (b) and dark‐field (c) radiographs can be simultaneously reconstructed with a perfect temporal and spatial registration. The attenuation‐based radiograph is equivalent to a conventional x‐ray radiograph, whereas the dark‐field image quantifies ultra‐small‐angle scattering, offering enhanced sensitivity to pulmonary microstructure beyond the resolution of conventional radiography.

The clinical dark‐field prototype, as described in Willer et al.^4^ and Gassert et al.,^10^ such as illustrated in Figure 1, records the attenuation and dark‐field images of patients. The system integrates a conventional x‐ray source (MRC 200 0508 ROT‐GS 1003, Philips Medical Systems) with an aluminum‐equivalent prefiltration of 2.5mm, a collimator box (R 302 MLP/A DHHS, Ralco), three gratings ($G_{0}$, $G_{1}$, and $G_{2}$), and a flat‐panel detector (PIXIUM 4343 F4, Trixell). The gratings are mounted on an interferometer arm, which is scanned in the vertical direction, as the gratings do not cover the entire vertical field of view. Moiré fringes are generated in the detector plane due to a slight periodic detuning of $G_{2}$. Moving the interferometer arm, a stepping curve is then measured for each pixel. A single scan comprises 195 exposures, producing 24 exposures for each pixel, sufficient to generate the stepping curve and so to extract the attenuation and dark‐field signals previously described by Equations (3) and (5). The total acquisition time for one image is approximately 7s. All images have been recorded with a tube voltage of 70kVp, a pulse rate of 30Hz, and a pulse duration of 17.1ms. For a more detailed description of the image reconstruction methodology, processing pipeline, and correction algorithms, the reader is referred to Refs. 6, 28 The detector features a 600‐μm‐thick CsI scintillator layer and operates with 3×3 pixel binning, resulting in a physical pixel size of 444μm. For study participants positioned at a contact plane 20cm from the detector, this corresponds to an effective pixel size of approximately 400μm in the posterior–anterior (p.‐a.) orientation. This value may vary slightly depending on patient size and positioning. For a reference patient scanned in the p.‐a. position, the effective dose is 35μSv.^7^ The tube current required to achieve the target detector dose of 3.75μGy at the scanning prototype system is determined for each individual study participant using a calibration curve and the dose information of the conventional system for COPD participants, and a body‐mass‐index (BMI) correlation curve for COVID‐19 participants.^29^

### 3.2 Selection of patient images

For the methods described below, radiographs taken at the fringe scanning system described in Section 3.1 were used. A total of 51 patient images classified as diseased from the COPD study, 86 from the COVID‐19 study, and 37 healthy patients from both studies were selected for the analysis of the BHC method presented here.

Spectral CT data were also acquired for two patients.

This retrospective study was conducted in accordance with the Declaration of Helsinki (as revised in 2013). Institutional Review Board approval was obtained from the Ethics Commission of the Medical Faculty, Technical University of Munich, Germany (reference numbers: 166/20S and 587/16S). Approval from the National Radiation Protection Agency was also secured prior to the initiation of the study. Written informed consent was obtained from all participants.

### 3.3 Segmentation

#### 3.3.1 Rib segmentation

Deriving and using a tissue map directly from the attenuation image is problematic because bone and soft tissue have different spectral properties, and bone regions always contain unknown mixtures of both, which affect the beam‐hardening‐induced dark‐field signal differently. It is therefore necessary to segment tissue into regions with and without bone contribution and to determine a representative weighting of bone and soft tissue. As a first step, automated rib segmentation was performed using a deep convolutional neural network based on a U‐Net architecture with skip connections and an EfficientNet‐B0 encoder pretrained on ImageNet. The model was trained on the publicly available VinDr‐RibCXR dataset,^30^ comprising 245 chest radiographs with manually annotated masks for the first 10 ribs bilaterally. The dataset was split into 196 training and 49 validation images.

A total of 16 attenuation radiographs acquired at the clinical dark‐field setup were manually annotated using an internal annotation tool to assess cross‐domain generalization of the rib segmentation model to images obtained with the dark‐field prototype. In each image, the first 10 ribs on both sides were labeled individually, resulting in 20 rib masks per image, consistent with the structure of the training data.

The network was implemented in PyTorch (v2.0.1) and trained using the Adam optimizer and Dice loss function on an NVIDIA RTX A4000 GPU. Training parameters are summarized in Table 1.

**TABLE 1:** Training parameters for rib and clavicle segmentation.

Model	Learning rate	Epochs	Batch size	# Classes
Rib	$1\times 10^{-3}$	195	16	20
Clavicle	$5\times 10^{-4}$	274	8	2

#### 3.3.2 Clavicle segmentation

Clavicle segmentation was conducted using a dataset of 80 attenuation images acquired with the clinical dark‐field scanner. Manual annotations were performed using the MATLAB segmentation toolbox (MathWorks, v9.14, R2023a). The dataset was stratified into 56 training, 12 validation, and 12 testing images.

The segmentation model employed the same U‐Net architecture and encoder as used for rib segmentation. The model was trained in PyTorch using the Adam optimizer and Dice loss. Training parameters were optimized for this task and are summarized in Table 1.

This segmentation model enables automated segmentation of the clavicles in attenuation images acquired from the dark‐field prototype, thereby supporting anatomically informed BHC. For both rib and clavicle segmentation models, segmentation performance was evaluated on internally annotated test data using Dice similarity coefficients, sensitivity, and specificity to verify sufficient anatomical localization for anatomically informed BHC.

### 3.4 Attenuation contribution mask creation

#### 3.4.1 Material decomposition

To derive material‐specific attenuation contributions, spectral raw data were acquired using a dual‐energy CT (IQon spectral CT, Philips Healthcare, Best, The Netherlands). Virtual Mono‐energetic images (VMIs) at 50 and 200keV were then generated for one male and one female patient using IntelliSpace Portal (version 12.1, Philips Healthcare). Using both VMIs of each patient, a material decomposition into water‐ and aluminum‐attenuation images was performed for each, serving as surrogates for soft tissue and bone, respectively. The water and aluminum attenuation maps were normalized to express relative contributions to total attenuation.

Cross‐sectional profiles through the ribs and clavicles in the decomposed attenuation maps were analyzed to derive region‐specific contribution values, differences between anterior and posterior ribs, and differences between male and female patients.

#### 3.4.2 Attenuation contribution mask generation

Beam hardening is caused exclusively by the absorbing materials, whereas lung tissue contributes only marginally and can be neglected. While individual patients naturally exhibit some variation in bone density, the purpose of the CT data analysis was to obtain an approximate estimate of the relative contributions of absorbing materials, namely bone and soft tissue. An exact patient‐specific determination would require spectral CT imaging, which is not routinely performed in clinical practice. Moreover, bone density in the rib region remains relatively stable even in the presence of osteoporosis,^31^ justifying the use of an approximate weighting. Anatomically resolved attenuation masks were generated by applying the attenuation contribution weights to the segmentation masks. Posterior and anterior rib components were separated by identifying the overlap region at the lung boundary; pixels superior to this boundary were assigned to posterior ribs, whereas those inferior were assigned to anterior ribs. To incorporate the increased attenuation at bone edges observed in spectral CT, a Sobel filter was applied to the binary masks to extract rib edges, which were subsequently dilated and incorporated into the attenuation map with a weighting factor of 0.05. The aluminum attenuation contribution map was smoothed using a Gaussian kernel (σ=2.2, truncated at 3σ) to ensure gradual spatial transitions and prevent sharp discontinuities in the correction process. The complementary water mask was obtained as shown in Equation (6).

(6) $$\begin{array}{cc} & \omega_{H_{2}O}\left(x,y\right)=1-\omega_{\mathrm{Al}}\left(x,y\right) \end{array}$$

The two most caudal ribs, not represented in the training data, were excluded from the segmentation. As these ribs primarily overlay abdominal structures, their omission was not expected to affect lung‐specific BHC.

#### 3.4.3 Beam‐hardening correction processing

The BHC procedure builds upon the single LUT method introduced by Lochschmidt et al., where the beam‐hardening‐induced dark‐field signal is corrected using LUTs for water and aluminum and averaged with a global aluminum weighting factor and a correction‐related bias correction term. Both have to be chosen depending on the clinical question. Since the bias correction term is theoretically dependent on how large the attenuation of pure water is in the image, but the exact position of these areas is not known, for example, in the form of a mask, a global value must be defined that is either a compromise of the entire attenuation range or only optimizes the correction for regions of equal attenuation. An optimization for the upper lung region would therefore lead to overcorrection in the heart region, and one needs to choose new parameters to optimize for the heart region, which would mean an undercorrection in the upper lung regions.

In the approach proposed here, this global weighting factor and the bias‐correction term are replaced with a spatially resolved weighting derived from attenuation contribution masks $\omega_{\text{Al}}\left(x,y\right)$ and 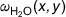 described above in combination with the deep‐learning‐based bone segmentation. Specifically, the aluminum and water masks are used to compute a pixel‐wise weighted sum of the LUT‐based beam hardening‐induced dark‐field signals $D_{p}^{\text{BH}}\left(x,y\right)$ (7). The resulting correction map $D_{p}^{\text{BH}}\left(x,y\right)$ is then subtracted from the raw dark‐field image $D_{p}\left(x,y\right)$ to obtain the corrected image 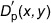 (8).

(7)

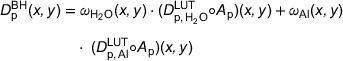



(8)

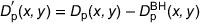

**FIGURE 2:**
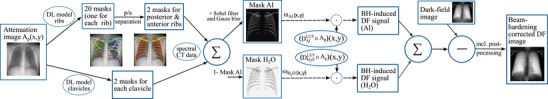
Schematic overview of the proposed beam‐hardening correction pipeline. Deep‐learning‐based models are used to segment ribs and clavicles from the attenuation image, followed by separation into anterior and posterior components. Material‐specific masks are generated using spectral CT‐based decomposition into aluminum and water. Beam‐hardening‐induced dark‐field signals are estimated via calibrated look‐up tables (LUTs) and refined through Gaussian smoothing and Sobel filtering. The resulting artifact maps are subtracted from the raw dark‐field image.

Figure 2 summarizes the complete pipeline, including segmentation of ribs and clavicles, p.‐a. separation, mask generation using spectral CT‐based attenuation contributions, and the final BHC implementation using pixel‐wise LUT interpolation and subtraction. The integration of this AI‐based automatic segmentation of relevant bone structures might improve the BHC by preventing regional overcorrections due to purely soft‐tissue structures. As a result, an additional bias term—as it was used in previous global correction algorithms—to correct for these overcorrections is no longer required.

### 3.5 Statistical analysis

All analyses were performed using Python (v3.8.10; NumPy v1.24.4, SciPy v1.10.1, pandas v2.0.3) and R (v4.5.0). To quantify the impact of the BHC, the sum of the dark‐field extinction coefficient was calculated for each patient's lung mask and finally normalized by the lung volume. The lung volume was calculated by a model using lateral and p.‐a. orientations of each patient.^32^

The effectiveness of BHC in improving the homogeneity of the lung dark‐field signal was evaluated using the coefficient of variation (CV) (9), the interquartile range (IQR) (10),^33^ and the overlap of two IQRs (11) computed within manually defined lung masks.

(9) $$\begin{array}{cc} & \text{CV}=\frac{\sigma }{\mu } \end{array}$$

(10) $$\begin{array}{cc} & \text{IQR}=Q_{3}-Q_{1} \end{array}$$

(11) $$\begin{array}{cc} & \delta_{\text{IQR(B)}}^{\text{IQR(A)}}=\max\left(0,\;\min\left(Q_{3}^{\left(A\right)},Q_{3}^{\left(B\right)}\right)\right.\\ & \left.-\max\left(Q_{1}^{\left(A\right)},Q_{1}^{\left(B\right)}\right)\right) \end{array}$$

where $\sigma^{2}$ is the variance and μ is the mean value. $Q_{1}$ separates the bottom 25% of the data from the rest and $Q_{3}$ the top 25% from the rest.

For each subject, CV and IQR were computed across the lung region in p.‐a. orientation, both before and after application of the segmentation‐based BHC. Lower values in these metrics correspond to increased signal homogeneity and thus indicate reduced beam hardening artifacts.

To assess the statistical significance of the CV reduction after BHC, the Wilcoxon signed‐rank test was applied to paired CV values (no BHC vs. BHC) within each clinical cohort (COPD, COVID‐19, Healthy). This nonparametric test does not assume normality and was used with a two‐sided significance threshold of p<0.05.

Group‐wise CV distributions were visualized using box plots. Percentage reductions were summarized using both mean and median values. The IQR was used descriptively to quantify signal spread.

## 4 RESULTS

### 4.1 Attenuation contribution mask

As described in Section 3.4.1, cross‐sectional profiles through the ribs and clavicles in the decomposed maps were performed and resulted in characteristic values for the posterior ribs, anterior ribs, clavicle, and the edges of the ribs. Furthermore, a uniform background contribution of 0.05 was measured in regions between ribs and incorporated into the weighting model. Table 2 summarizes the empirically derived aluminum weights.

**TABLE 2:** Aluminum weights for several bone regions and the background.

Aluminum	Posterior	Anterior	Clavicle	Edge	Background
Male	0.15	0.1	0.2	0.05	0.05
Female	0.1	0.05	0.2	0.05	0.05

### 4.2 Qualitative evaluation

**FIGURE 3:**
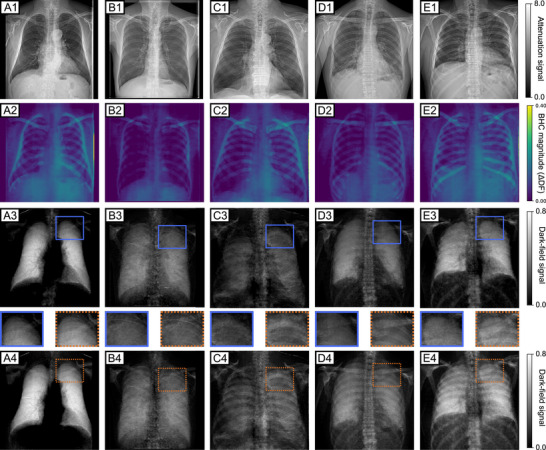
Effect of segmentation‐based beam hardening correction (BHC) across representative clinical cases. Five representative patients are shown, including one healthy subject (a), two patients with chronic obstructive pulmonary disease (COPD) of differing severity (b, c), and two patients with COVID‐19 pneumonia (d, e). Row 1 (a1–e1): attenuation‐based conventional radiographs. Row 2 (a2–e2): Beam‐hardening correction magnitude, computed as the difference between uncorrected and corrected dark‐field radiographs. Row 3 (a3–e3): Dark‐field radiographs with applied proposed beam‐hardening correction. Row 4 (a4–e4) Dark‐field radiographs without beam‐hardening correction. In the uncorrected dark‐field radiographs, pronounced rib‐ and clavicle‐induced artifacts are visibile and obscure the lung parenchyma. Insets highlight representative regions affected by beam‐hardening artifacts before correction **(orange dashed boxes)** and the corresponding regions after correction **(orange blue boxes)**. The attenuation‐based conventional radiographs and beam‐hardening correction magnitude illustrate patient‐specific differences in bone attenuation and overlap with lung tissue. Following segmentation‐based BHC, rib‐ and clavicle‐induced artifacts are substantially reduced across all cases, yielding a more homogeneous dark‐field signal.

The proposed segmentation‐based BHC described was applied to 174 clinical dark‐field chest radiographs from patients not included in the segmentation model's training or validation cohorts. Representative results from five patients—one healthy subject, two with COPD of varying severity, and two with COVID‐19 pneumonia—are shown in Figure 3. For each representative case shown in Figure 3, the attenuation‐based conventional radiograph, the uncorrected and corrected dark‐field images, and the corresponding BHC magnitude are displayed. Across all representative cases, the BHC magnitude spatially coincides with regions of increased rib and clavicle attenuation, while visible differences between uncorrected and corrected dark‐field images are apparent.

**FIGURE 4:**
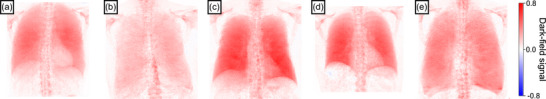
Visualization of the absence of overcorrections. (a)–(e) show five additional study participants corrected using the AI‐based beam hardening correction (BHC). To demonstrate the absence of overcorrection, the images are displayed using a colormap in which white corresponds to a dark‐field signal of 0.0. Regions consisting exclusively of soft tissue are fully and uniformly corrected. Areas in which bone structures are visible correspond to anatomical regions that do not contribute to the lung dark‐field signal. These regions were excluded from the spectral measurements in the original analysis.

One of the main reasons for integrating AI‐based rib segmentation into the LUT‐based correction was the elimination of overcorrection artifacts due to beam hardening. To demonstrate this, colormaps are shown for five representative patients (Figure 4), which clearly show that overcorrection is effectively resolved by incorporating rib segmentation.

In the uncorrected images, pronounced beam‐hardening‐induced signals from osseous structures, particularly ribs and clavicle, are visible across the lung. These structured artifacts introduce marked inhomogeneity and obscure the overlapping lung parenchyma. Following BHC, these bone‐induced features are substantially reduced, yielding a more homogeneous signal distribution and improving visualization of the pulmonary microstructure. Insets highlight the segmented rib and clavicle regions, further illustrating artifact suppression by the BHC. As an additional validation of the segmentation step used in the proposed pipeline, segmentation accuracy was evaluated on internally annotated test data. The rib and clavicle segmentation models achieved mean Dice similarity coefficients of 0.83±0.06 and 0.93±0.04, respectively, indicating sufficient accuracy for generating the attenuation contribution masks required for segmentation‐based BHC.

### 4.3 Quantitative evaluation

**FIGURE 5:**
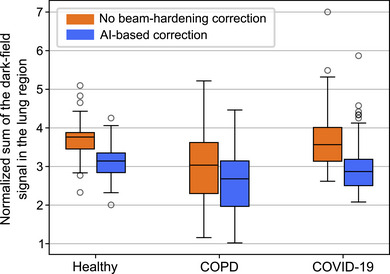
Quantitative comparison of beam hardening correction (BHC) approaches in clinical dark‐field chest radiography. The plot shows the sum of the dark‐field signal in the lung region, normalized by lung volume, for three patient cohorts: healthy, chronic obstructive pulmonary disease (COPD), and COVID‐19. Results are shown without a correction of beam‐hardening effects (orange) and with the proposed AI‐segmentation‐based BHC (blue). The segmentation‐based approach results in consistently higher normalized dark‐field signals across all cohorts, indicating improved recovery of true lung signal and reduction of beam hardening artifacts from bones. Each box indicates the interquartile range with the median, and whiskers extend to 1.5× the IQR; outliers are shown as circles. The statistical evaluation described in 3.5 is shown in Table 3.

Group‐wise comparisons were performed across healthy, COPD, and COVID‐19 cohorts by assessing the total volume obtained from the normalized sum of the dark‐field signal described in Section 3.5. Figure 5 shows the resulting distributions for the uncorrected signal and the proposed segmentation‐based BHC.

Across all cohorts, segmentation‐based BHC resulted in a lower normalized dark‐field signal. In healthy subjects, the mean signal decreased from 3.76 to 3.14 (−16.5%), in COPD patients from 3.04 to 2.68 (−11.8%), and in COVID‐19 patients from 3.57 to 2.87 (−19.6%).

In addition to reductions of the normalized dark‐field signal, diagnostic class separability improved. The median overlap between healthy and COPD patients decreased from 2.77 to 2.25 (−18.8%), and between healthy and COVID‐19 patients from 2.48 to 2.17 (−12.5%). Both reductions were statistically significant (p<0.01, Wilcoxon signed‐rank test).

To further evaluate intrapatient signal homogeneity, the CV was computed individually for each patient based on their lung segmentation. As shown in Table 3, segmentation‐based BHC leads to a consistent and significant CV reduction across all three cohorts. In healthy subjects, CV decreased from 0.32 to 0.27; in COPD patients, from 0.31 to 0.26; and in COVID‐19 patients, from 0.38 to 0.31. All changes were statistically significant (p<0.001, Wilcoxon signed‐rank test), indicating improved spatial uniformity of the lung dark‐field signal. As an additional validation of the segmentation step within the proposed pipeline, segmentation accuracy was evaluated on internally annotated test data. The rib and clavicle segmentation models achieved mean Dice similarity coefficients of 0.83±0.06 and 0.93±0.04, respectively, indicating sufficient anatomical accuracy for the generation of attenuation contribution masks used in the segmentation‐based BHC.

**TABLE 3:** Statistical analysis of the AI segmentation‐based method in Section 3.5.

Parameters	${\tilde{x}}_{\text{Healthy}}$	${\tilde{x}}_{\text{COPD}}$	${\tilde{x}}_{\text{COVID-19}}$	$\delta_{\text{IQR(COPD)}}$ 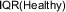	$\delta_{\text{IQR(COVID-19)}}$ 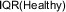	${\mathrm{CV}}_{\text{Healthy}}$	${\mathrm{CV}}_{\text{COPD}}$	${\mathrm{CV}}_{\text{COVID-19}}$
Values (no BHC)	3.76	3.04	3.57	2.77	2.48	0.32	0.31	0.38
Values (DL‐Seg)	3.14	2.68	2.87	2.25	2.17	0.27	0.26	0.31
**Relative change**	−16.5%	−11.8%	−19.6%	−18.8%	−12.5%	−15.6%	−16.1%	−18.4%
Wilcoxon test (*p*‐value)	—	—	—	<0.01	<0.01	<0.01	<0.01	<0.01

*Note*: $\tilde{x}$ denotes the median; CV per Equation (9); IQR‐overlap per Equation (11).

## 5 DISCUSSION

In grating‐based dark‐field radiography, highly attenuating anatomical regions such as ribs and clavicles introduce artifacts due to the beam‐hardening‐induced dark‐field signal. These artifacts obscure lung parenchyma and reduce both the interpretability and quantitative reliability of dark‐field chest radiographs.

To address this limitation, a segmentation‐based BHC framework was developed and evaluated in a clinical setting. The method combines deep‐learning‐based anatomical segmentation of osseous structures and material‐specific attenuation weighting derived from dual‐energy spectral CT decomposition with LUT‐based methods published before. This enables regionally adaptive correction of beam‐hardening‐induced dark‐field signal and prevents overcorrections within the entire dark‐field images while preserving true microstructural signals from lung tissue. Although the absolute ground truth of the lung dark‐field signal is not directly measurable, it has already been shown mathematically that the signal obtained by subtracting a LUT‐based approach is purely beam‐hardening‐related. Anatomically, well‐defined intercostal regions provide a reliable reference, as they contain only soft tissue and water equivalent material. At rib boundaries, deviations from the intercostal signal indicate under‐ or overcorrection, while the absence of edge artifacts suggests accurate correction, consistent with the anatomical continuity of lung parenchyma. While a formal diagnostic task analysis was beyond the scope of this work, the observed reduction of rib‐induced artifacts and improved signal homogeneity are essential prerequisites for reliable quantitative analysis and downstream clinical applications. Nevertheless, the improved visualization needs to be validated in future reader studies.

Applied to a clinical cohort of 174 patients, the proposed method demonstrated consistently improved image quality and quantitative characteristics across healthy subjects and patients with chronic COPD and COVID‐19 pneumonia.

The correction significantly reduced rib‐ and clavicle‐induced artifacts, yielding more homogeneous dark‐field representations of the lungs and improving the visibility of parenchymal regions. Quantitative analysis confirmed a statistically significant reduction in volume‐normalized dark‐field signal variation across all cohorts. More importantly, diagnostic separability improved: distributional overlap between healthy and COPD patients decreased by 18.8%, and between healthy and COVID‐19 patients by 12.5% (both p<0.01, Wilcoxon signed‐rank test). These findings support improved classification potential for disease detection and differentiation. Although the correction reduces absolute mean dark‐field signal levels, disease separability improves due to the suppression of artifact‐induced variability and reduced distributional overlap, rather than increased absolute signal contrast.

Beyond intergroup discrimination, the method also improved intrapatient signal consistency markedly. The CV within individual lung masks decreased significantly across all cohorts (p<0.01), indicating enhanced spatial homogeneity of the corrected dark‐field signal.

The observed reduction in spatial variability is attributable to the selective suppression of anatomically correlated beam‐hardening artifacts, rather than to a uniform rescaling of the dark‐field signal. The correction also increased the contrast between healthy lung parenchyma and diseased lungs, potentially improving sensitivity in early disease detection.

Several limitations of the proposed framework should be acknowledged, as they define the current scope of the method's clinical applicability. The performance of the correction is intrinsically dependent on the accuracy of the deep‐learning‐based segmentation, since segmentation errors propagate into the attenuation contribution masks and may lead to local over‐ or under‐correction. This dependency is particularly relevant in cases with strongly altered anatomy, where segmentation performance can degrade. The internal segmentation validation data were used to assess robustness of the segmentation framework for anatomically informed BHC. Furthermore, the two most caudal ribs were excluded from the segmentation due to the lack of corresponding training annotations. As these ribs can partially overlap the basal lung regions, residual beam hardening effects can persist locally in the lower lung lobes. In addition, metallic implants and foreign bodies are not addressed by the current two‐material model and segmentation framework and may therefore induce uncorrected artifacts. While these aspects limit the generality of the present implementation, the proposed method consistently improves bone‐induced artifact suppression in clinical dark‐field chest radiographs.

Future work should explore the integration of spectral imaging into the clinical dark‐field system. Dual‐layer, photon‐counting detectors or rapid kVp‐switching systems can provide direct, pixel‐wise material decomposition without reliance on segmentation. These detectors or acquisition types enable the acquisition of spectrally resolved x‐ray data, allowing basis‐material decomposition into soft tissue and bone or water and aluminum, respectively. Such an approach would enable fully patient‐specific correction and eliminate dependence on anatomical priors or training data. Prior work has demonstrated the feasibility of spectral models for dark‐field signal characterization,^34^, ^35^ including differentiation of diseases such as emphysema and fibrosis.^36^ Integrating these models into the correction pipeline could further improve robustness, generalizability, and clinical applicability.

## 6 CONCLUSION

This study presents the first fully automated, anatomically adaptive BHC framework for clinical x‐ray dark‐field chest radiography. The proposed method combines deep‐learning‐based segmentation of ribs and clavicles with material‐specific attenuation weighting to suppress beam hardening artifacts introduced by osseous structures. The correction improves intrapulmonary signal homogeneity, enhances diagnostic separability between healthy and diseased lungs, and supports both qualitative and quantitative interpretation of dark‐field images. These improvements address a major limitation of dark‐field imaging and support its further integration into clinical research and diagnostic workflows.

## CONFLICT OF INTEREST STATEMENT

Thomas Koehler is an employee of Philips Innovative Technologies, Hamburg, Germany. The other authors declare no conflicts of interest.

## Data Availability

The data underlying this study are available upon reasonable request from the corresponding author, L. Kaster (lennard.kaster@tum.de).
